# Efficacy of Isoniazid–Rifampicin Preventive Therapy in Adolescent Contacts in School Outbreaks: A Retrospective Cohort Study in Eastern China

**DOI:** 10.3390/pathogens14121203

**Published:** 2025-11-26

**Authors:** Zhan Wang, Rong Wang, Wenjin Wang, Wenxin Jiang, Xinru Fei, Jingxian Ning, Yuchen Pan, Limei Zhu, Wei Lu, Qiao Liu

**Affiliations:** 1 Department of Chronic Communicable Disease, Center for Disease Control and Prevention of Jiangsu Province, Nanjing 210009, China; 18252103539@163.com (Z.W.); wenxin001102@163.com (W.J.); feixinru0813@163.com (X.F.); ning329595@163.com (J.N.); lilyam0921@163.com (L.Z.); 2 Department of Epidemiology, Center for Global Health, School of Public Health, Nanjing Medical University, Nanjing 211166, China; luwei@jscdc.cn; 3 Department of Chronic Communicable Disease, Nanjing Municipal Center for Disease Control and Prevention, Nanjing 210003, China; wrnjmu@163.com; 4 Department of Communicable Disease, Center for Disease Control and Prevention of Yancheng City, Yancheng 224000, China; jww970805@163.com; 5 Department of Epidemiology, School of Public Health, Southeast University, Nanjing 210009, China; panapan0011@163.com

**Keywords:** tuberculosis preventive therapy, adolescent contacts, latent tuberculosis infection, school outbreaks

## Abstract

Adolescents are underprioritized in tuberculosis (TB) control, and the effect of TB preventive therapy (TPT) in this group is unstudied in China. This study evaluated the protective effect of TPT in Chinese adolescents during TB outbreaks. Data on TB outbreaks and contact screening in six cities (2019–2021) were collected. Adolescents eligible for TPT were identified via tuberculin skin test or interferon-gamma assay and grouped by TPT. Follow-up until 31 December 2023, tracked TB onset. The protective effect was analyzed using KM survival curves and COX models. From January 2019 to December 2021, 136 school TB outbreaks were reported, involving 10,837 adolescent contacts. Among these, 624 adolescent contacts met the criteria for TPT (latent TB infection) at baseline; 277 (44.4%) initiated a 3-month isoniazid plus rifampicin preventive therapy (TPT group), while 347 (55.6%) did not receive TPT (non-TPT group). By 31 December 2023, 11 of these 624 adolescent contacts developed active TB, with 1 patient in the TPT group and 10 patients in the non-TPT group. The cumulative incidence of TB was 0.36% in the TPT group vs. 2.88% in the non-TPT group (χ^2^ = 5.65, *p* = 0.017). This corresponds to an approximate 87% reduction in TB incidence among adolescent contacts who received TPT compared to those who did not. TPT reduced TB incidence by ~90% among adolescent contacts. Timely, comprehensive, and standardized TPT is recommended to minimize TB risks in educational settings and achieve a TB-free campus.

## 1. Background

Tuberculosis (TB) is a chronic infectious disease caused by *Mycobacterium tuberculosis* (*M.tb*) infection. According to the latest report of the World Health Organization, there are 10.8 million new patients of TB in 2023, which has become a major global public health problem threatening human health. China is one of the 30 countries with a high burden of TB in the world, with about 741,000 new patients of TB in 2023, accounting for 6.8% of the global total and ranking third in the world [1]. In 2014, WHO put forward the “END TB” Strategy, which aims to reduce TB mortality by 95% and morbidity by 90% by 2035 [2,3]. Approximately 1.8 million adolescents develop TB every year [4]. In recent years, the incidence of TB among students has shown a rising trend across in China [5].

Latent tuberculosis infection (LTBI) is a condition in which the body is continuously immune to *M.tb*, with no host symptoms, no lesions, and no clinical evidence of active TB [6]. Among adolescents with latent TB infection, 10% to 15% may develop the disease [7]. In addition, studies have shown that adolescence is a period of increased susceptibility to TB, when latent TB infection rates and TB incidence rise significantly [8,9]. Adolescents with *M.tb* infection have significantly greater chance of progressing to TB compared to their younger age counterparts from 5–10 years of age [10,11].

It is of great significance to carry out tuberculosis-preventive treatment (TPT) of LTBI to reduce the morbidity of students [12,13]. The results of a survey [14] showed that the cumulative one-year incidence of LTBI in adolescents without TPT was 268.17 per 100,000, and that of adolescents with preventive treatment was 51.12 per 100,000. A study followed for 18 years found that the incidence of active TB among adolescents dropped from 25.9 per 100,000 adolescents to 10.9 per 100,000 through preventive treatment [15].

However, there is a lack of research on the effect of TPT on adolescents in China, particularly in terms of real-world research outcomes derived from large sample sizes. In this study, data on adolescents taking TPT during school outbreaks were collected, and the protective effect of a 3-month combination of isoniazid and rifampicin in adolescent contacts was analyzed in order to provide a scientific basis for the prevention and control of TB in schools.

## 2. Methods

### 2.1. Setting and Recruitment

The research was conducted across middle schools located in Lianyungang, Yancheng, Nanjing, Suzhou, Zhenjiang, and Xuzhou city of Jiangsu Province. Adolescents in this study comprised adolescents aged 12–20 years who had documented close contact exposure to active tuberculosis patients during outbreak investigations conducted between January 2019 and December 2021. To ensure the reliability and validity of the findings, specific inclusion criteria were meticulously established. These criteria mandated that adolescent contacts not only engaged in the survey but also completed it in its entirety. Furthermore, all adolescent contacts were required to undergo a validated test for LTBI as part of the outbreak contact screening process.

This study was approved by the ethics committee of Jiangsu Center for Disease Control and Prevention. Written informed consent was obtained from study adolescent contacts (JSJK2022-B010-01). Prior to these screenings among students, a notification was issued that required signatures and consent from both students and parents.

### 2.2. Screening of Contacts

Tuberculin skin test (TST) results were obtained through the Mantoux method, as elaborated in previous studies. Specifically, 0.1 mL of 5 tuberculin units of purified protein derivative (PPD) was carefully injected intradermally in the left forearm. After the injection, experienced nurses, well-trained in reading such tests, evaluated the tuberculin skin test results 48 to 72 h later. They measured the average longitudinal and transverse diameters of the PPD-induced induration, which were taken as the average diameters of induration, following the standard protocol [16] For adolescents who were not suitable for TST testing, such as those with certain skin conditions, a sensitive interferon gamma assay (IGRA) was used to screen for LTBI.

### 2.3. Tuberculosis Preventive Treatment

In this study, we defined latent tuberculosis infection as those with a TST ≥ 10 mm or more, or those with a positive IGRA result [17]. However, TPT was given to LTBI with a ≥15 mm according to National School TB Prevention and control guidelines [18]. Prior to initiating TPT, all eligible contacts underwent comprehensive clinical evaluation to exclude active TB disease. This screening protocol included clinical symptom assessment, chest radiography, and sputum examination when indicated. Contacts with any clinical, radiological, or bacteriological evidence of active TB disease were excluded from TPT and referred for further diagnostic workup. The TPT regimen consisted of isoniazid combined with rifampicin for a total duration of three months (3HR), administered under standardized dosing protocols. Throughout the TPT period, participants were monitored regularly for adverse drug reactions. Upon completion of the 3HR regimen, a formal treatment closure evaluation was performed.

### 2.4. Incident Tuberculosis Disease

All adolescent contacts in this study were integrated into a comprehensive data linkage with the provincial TB surveillance system, which is maintained by the corresponding six municipal Centers for Disease Control and Prevention (CDC). In China, TB is classified as a reportable disease, necessitating that all adolescent contacts diagnosed with TB are systematically managed and monitored through the TB Management Information System at the city, municipal, and provincial levels.

The data linkage encompassed weekly records spanning a five-year period, from January 2019 to January 2024. Adolescent contacts were meticulously linked and matched to the Tuberculosis Management Information System (TBMIS) using a multi-faceted approach, leveraging several key identifying variables. These included full name (first and last), date of birth, age, sex, and residential address. This rigorous matching process minimized the risk of misidentification and ensured accurate tracking of TB patients within the cohort.

All adolescent contacts diagnosed with TB in our study were confirmed through bacteriological evidence or clinical diagnosis. For each adolescent contact, the time-to-event was defined as the duration from the start of the follow-up period until the diagnosis of tuberculosis. Those who remained free of active TB were followed until the end of the study period (31 December 2023). In cases where an adolescent contact was identified as having TB, additional detailed information was gathered to provide a comprehensive overview of their medical condition. This included an examination of their medical records, results from chest radiological examinations, bacteriological test outcomes, drug sensitivity test results, prescribed treatment regimens, and treatment outcomes.

## 3. Statistical Analysis

Continuous variables were summarized using interquartile ranges (IQRs), providing a robust measure of central tendency and dispersion that is less sensitive to outliers than the mean and standard deviation. Categorical variables, on the other hand, were presented using standard 2 × 2 contingency tables, allowing for a clear visualization of the distribution of adolescents across different categories. To compare the frequencies of categorical variables between groups, either the Pearson chi-squared (χ^2^) test or the Fisher exact test was employed, depending on the sample size and expected cell counts within the contingency tables. The Fisher exact test was used when the assumptions of the chi-squared test were not met, particularly when dealing with small sample sizes or low expected frequencies. For these variables, mean imputation was employed to handle missing values, ensuring complete case representation in descriptive analyses.

The longitudinal aspect of the study involved a five-year follow-up period, from January 2019 to December 2023, to monitor the development of TB among adolescent contacts. For each participant, the time-to-event was defined as the duration from the start of the follow-up period until the diagnosis of TB. TB incidence was calculated and expressed as the number of new patients per 100,000 person-years, providing a standardized measure of disease occurrence within the study population. Incidence rate ratios (IRRs) were also calculated to compare the incidence of TB between different groups, along with corresponding 95% Poisson confidence intervals (CIs) to quantify the precision of these estimates.

To further investigate the factors associated with the risk of developing TB, Cox proportional hazards models were utilized for multivariable analysis. This method allowed for the assessment of the relationship between multiple independent variables and the time-to-event outcome (TB diagnosis), while controlling for the influence of potential confounders. The results of these models are presented as hazard ratios (HRs), which represent the relative risk of developing TB associated with a one-unit change in the independent variable. For each independent variable, both univariable and multivariable models were constructed. Univariable models examined the crude association (indirect effect) between the independent variable and TB risk, while multivariable models assessed the adjusted association (direct effect) after accounting for the effects of other relevant variables, thus providing a more accurate estimate of the independent variable’s true impact on TB risk. Variables with a univariable *p* < 0.05 were included in the multivariable Cox model, and sex and age were additionally forced into the model as demographic confounders, regardless of their statistical significance. We also analyzed risk factors for developing active TB among adolescent contacts who had LTBI but did not receive preventive treatment. Furthermore, using univariable Cox models, HRs at 3 months, 1 year, 2 years, and 3 years of follow-up were calculated to assess how the association between each predictor and TB risk evolves over time.

## 4. Results

### 4.1. Study Population

**Figure 1:**
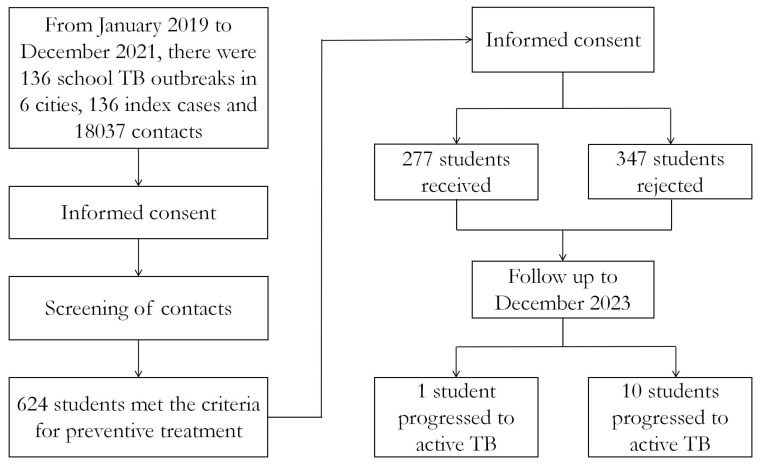
Flowchart of adolescent contacts enrolled in follow-up cohort for incident tuberculosis.

Between January 2019 and December 2021, six cities reported a total of 136 school-based TB outbreaks, with annual distributions of 38 in 2019, 53 in 2020, and 45 in 2021. These outbreaks involved 136 index patients and 10,837 close contacts. Among the contacts screened, 616 tested strongly positive for TST results and 8 positive for IGRA-positive. A total of 624 adolescent contacts met the criteria for TPT; 277 (44.4%) received treatment, while 347 (55.6%) did not. As of 31 December 2023, 21 adolescent contacts had progressed to active TB, 11 of whom were among those earmarked for preventive therapy at baseline screening (Figure 1).

### 4.2. Baseline of Index Patients and Contacts

Among 136 index patients, 74 were male, accounting for 54.4%. The highest annual patient count occurred in 2020, reaching 53 (39.0%). There were more bacteriological positives in new patients (58/63) and rifampicin-sensitive patients (58/63) (Table 1). Among the 10,837 adolescent contacts, 6090 were male, accounting for 56.2%. Yancheng city had the highest proportion (26.5%) and the highest number of contacts in 2020, reaching 3959 (36.5%). The majority of contacts (84.7%) showed no evidence of LTBI. Nearly all contacts (99.5%) underwent TST screening (Table 2).

### 4.3. Characteristics of Tuberculosis Secondary Patients During Follow-Up Time

Among the 21 secondary patients, 10 were male, accounting for 47.6%. The proportion from Yancheng city was the highest (27.3%), and the proportion from Nanjing city was the lowest (only 4.5%). The highest number of patients occurred in 2019, with 11 patients (52.4%). The numbers of bacteriologically negative and positive patients were similar (11 and 9), and most patients with positive bacteriology were sensitive to rifampicin (7/9). All patients were new patients and had no complications. One patient was resistant to rifampicin, and most patients (76.2%) were treated with a 6-month regimen of 2HRZE/4HR. In the end, 7 (33.3%) were cured, 12 (61.9%) completed the course, and 1 was transferred to MDR-treatment (Supplementary Table S1).

Among the 11 secondary patients who met the treatment criteria at baseline, 4 were male, accounting for 36.4%. The proportion from Xuzhou was the highest (36.4%), and the proportion from Zhenjiang was the lowest (only 9.09%). The highest number of patients occurred in 2020, with eight patients (72.7%). All patients were new patients and had no complications. Most patients (81.8%) were treated with a 6-month regimen of 2HRZE/4HR. In the end, 3 (27.3%) were cured and 12 (72.7%) completed the regimen (Table 3).

### 4.4. Protective Effect of Preventive Treatment for Students

**Figure 2:**
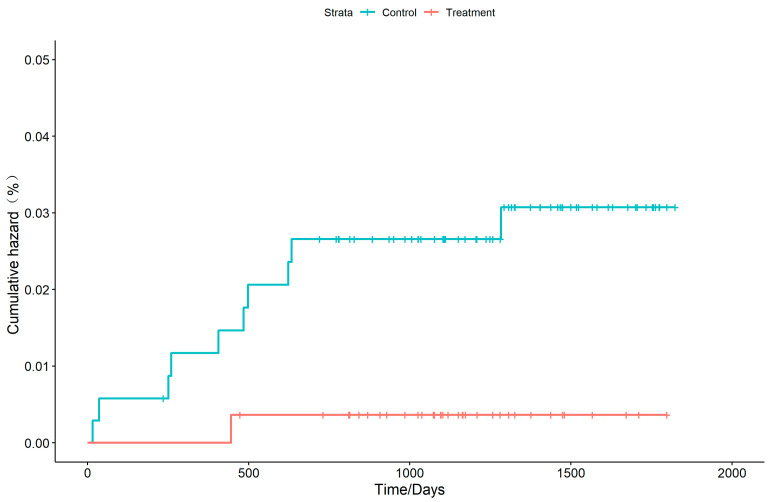
Risk of developing active tuberculosis among adolescent contacts with latent tuberculosis infection.

The adolescent contacts who received TPT were monitored for a total of 1088 person-years, during which one student developed active TB. Conversely, the non-TPT contacts were observed for 1268 person-years, resulting in 10 patients of active TB. The cumulative incidence of active TB was found to be 0.36% in the TPT group and 2.88% in the non-TPT group, with a statistically significant difference between the two groups (χ^2^ = 5.65, *p* < 0.05). The TPT exhibited a protection rate of 87.47%. The survival curve analysis indicated a significantly higher cumulative incidence in the non-TPT group compared to the TPT group, with a statistically significant difference (*p* < 0.05) (Figure 2).

### 4.5. Analysis of Factors Influencing the Effect of TPT

Univariate Cox analysis revealed that being a retreatment patient (HR = 3.599, 95% CI: 1.050 −12.329) and receiving preventive treatment (HR = 0.124, 95% CI: 0.016–0.969) were both significantly associated with the development of active TB. For other time points, there were no significant results (Supplementary Table S2). Subsequently, significant variables identified in the univariate analysis were included in the multivariate analysis, which demonstrated that the contacts of retreatment index patients (HR = 5.317, 95% CI: 1.542–18.340) were at increased risk for morbidity, while those receiving TPT (HR = 0.086, 95% CI: 0.011–0.678) were associated with decreased risk of morbidity (Supplementary Table S3). Contact with retreatment index patients was a risk factor for developing active TB among students who had LTBI but did not receive TPT (HR = 5.203 95% CI: 1.456–18.588) (Supplementary Table S4).

## 5. Discussion

Between 2019 and 2021, there were a total of 136 school TB outbreaks reported in six cities. Yancheng City had the highest number of patients (41), followed by Zhenjiang City (27), and Nanjing City, with the lowest number of patients (6). The year 2020 accounted for the highest number of patients, with 53 reported patients (39.0%). The cumulative incidence rates were 0.36% in the group receiving treatment, 2.88% in the group not receiving treatment, and a preventive treatment protection rate of 87.47%. The findings from a 10-year longitudinal study [19] evaluating the efficacy of isoniazid preventive therapy in a cohort of college students with strongly positive tuberculin skin tests revealed a preventive treatment efficacy rate of 63.48%, lower than the results reported in our own investigation. Previous research has indicated that the risk of developing active TB following Mycobacterium TB infection escalates steadily from the age of 12 to 19 years [20]. In addition, different preventive treatment regimens may also contribute to the difference in protection rates.

In this study, two primary patients were found to be linked to three secondary patients, respectively. Educational institutions, such as schools, are environments characterized by high population density, where students congregate for extended periods, fostering conditions conducive to the transmission of Mycobacterium TB. This is combined with heightened academic demands, resulting in limited opportunities for physical activity, inadequate rest, and compromised immune function, thereby increasing the likelihood of progression from latent TB infection to active TB. This underscores the significance of timely detection, as research indicates that screening contacts for TB after the onset window, even if promptly initiated, does not prevent outbreaks. Schools should routinely arrange health assessments for students to promptly identify TB patients and latent infections among contacts, enabling timely preventive measures to reduce the incidence.

The retreatment patient indicator was identified as a risk factor for disease among contacts. According to a study in Xinjiang’s Kashgar prefecture [21], initially treated TB is a risk factor for close contacts. This is inconsistent with our findings, possibly due to more health education among patients undergoing rehabilitation and a decline in the proportion of patients undergoing rehabilitation due to measures such as supervised medication for TB patients in the region. Retreaters take longer to diagnose than initial patients [22]. The lesion area may be larger, or a new lesion may appear [23]. They also have poorer physical health [24] increasing the risk of illness in the contacts [25]. It is recommended that more attention is paid to contacts of retreated patients, as retreated patients have a higher rate of drug resistance [26]. If a contact is infected with resistant bacteria, the harm to their health and the cost of treatment will increase significantly. The results of a study in Beijing, which showed that the index patients were etiologically positive TB patients were at higher risk of developing the disease among their contacts, are inconsistent with our findings. This may be due to the fact that the Beijing study involved boarding students, who were more likely to expel bacteria and have a higher risk of developing disease among their contacts than those who were negative, and that the same dormitory environment increased the chance of infection. Our study found that most of the characteristics of the index patients were unrelated to the onset of contact disease, indicating that in the event of a school TB epidemic, contact screening should be carried out for the index patients, regardless of age, etiology results, and other characteristics, and should not be taken lightly.

This study systematically describes the preventive treatment of middle school students with latent TB infection in Jiangsu Province and analyzes the factors affecting the protective effect, which fully proves the necessity of preventive treatment of middle school students with latent TB infection. However, there are some limitations. First, almost all latent infections are judged based on TST, and the sensitivity of the tuberculin skin test is between 68% and 95% [27]. The disadvantage is that the specificity is low and it is difficult to distinguish between BCG vaccination and NTM infection. The IGRA test is expensive and invasive, and it is not suitable for use in large population bases or initial screenings [28]. Previous studies have shown that people who test positive for tuberculin skin but negative for gamma-IFN release have a significantly higher incidence of both tests being positive [29]. In the case of a school TB outbreak, the initial screening recommended is the tuberculin skin test, and then the gamma-IFN release test for moderately strong positive patients, which is not only economical and convenient, but also improves the specificity [30]. Secondly, this study is a retrospective analysis, and there may be data missing. Finally, some students were excluded from the study due to a lack of interview records, which may have some influence on the conclusions of the study.

## 6. Conclusions

This study, based on data from a substantial cohort sample, demonstrates the efficacy of prophylactic medication in significantly reducing the incidence of TB among students, potentially mitigating up to 90% of the associated risk. Upon detection of a TB patient, it is recommended to immediately conduct comprehensive contact investigation and screening, promptly initiate TPT for all eligible close contacts, and implement integrated infection control measures to prevent further transmission.

## Figures and Tables

**Table 1:** Baseline characteristics of tuberculosis index patients in this study.

Characteristics	N	Bacteriologically Positive	Bacteriologically Negative or No Results	χ^2^/t	*p*-Value
Sex				1.74	0.19
Male	74	31	43		
Female	62	33	29		
Mean age, years (SD)	15.95 ± 1.74	15.73 ± 1.74	16.14 ± 1.74	−1.25	0.21
City				6.62	0.25
Lianyungang	20	7	13		
Yancheng	41	19	22		
Nanjing	6	3	3		
Suzhou	25	17	8		
Zhenjiang	27	12	15		
Xuzhou	17	6	11		
Year				3.11	0.21
2019	38	16	22		
2020	53	22	31		
2021	45	26	19		
Patient type				0.01	0.91
New patients	125	59	66		
Retreatment patients	11	5	6		
Complication				1.80	0.18
No	134	64	70		
Yes	2	0	2		
Treatment regimens				2.74	0.26
2HRZE/4HR	109	49	60		
2HRZE/10HR	4	1	3		
Other regimens	23	14	9		
Treatment outcome				105.941	<0.01
Cure	57	55	2		
Completing the treatment	76	6	70		
Death	1	0	1		
Adverse reaction	1	1	0		
Transfer to RR-TB therapy	1	0	1		

SD, standard deviation; HRZE, isoniazid, rifampicin, pyrazinamide, and ethambutol; HR, isoniazid and rifampicin.

**Table 2:** Demographic characteristics of 10,837 adolescent contacts of tuberculosis patients, stratified by the eligibility and administration of TPT.

	All Contacts	Received TPT	Eligible but Did Not Receive TPT	Not Eligible for TPT
Contact characteristics				
Median age, years (IQR)	16.00 (±2.00)	16.00 (±2.00)	16.00 (±2.00)	16.00 (±2.00)
Mean age, years (SD)	16.23 ± 1.58	16.68 ± 1.57	16.54 ± 1.57	16.21 ± 1.58
Age group, years				
<15	1345	21	28	1296
15–16	4530	84	125	4321
17–18	4433	151	168	4114
19 and above	529	21	26	482
Sex				
Male	6090	154	176	5760
Female	4747	123	171	4453
Year of outbreak				
2019	3472	136	115	3221
2020	3959	85	175	3669
2021	3406	56	57	3293
City				
Lianyungang	1801	55	110	1636
Yancheng	2867	52	76	2739
Nanjing	282	10	3	269
Suzhou	975	0	14	961
Zhenjiang	2312	145	65	2102
Xuzhou	2600	15	79	2506
Screening method				
TST	10,783	270	346	10,167
IGRA	54	7	1	46
Index case characteristics				
Median age, years (IQR)	16.00 (±2.00)	15.00 (±1.00)	16.00 (±2.00)	16.00 (±2.00)
Mean age, years (SD)	15.86 ± 1.53	15.65 ± 1.53	16.00 ± 1.53	15.86 ± 1.53
Age group, years				
<15	1634	29	23	1582
15–16	5512	187	197	5128
17–18	3253	49	120	3084
19 and above	438	12	7	419
Sex				
Male	5514	186	157	5171
Female	5323	91	190	5042
Patient type				
New patients	9372	159	268	8945
Retreatment patients	1465	118	79	1268
Treatment outcome				
Cure	4515	216	109	4190
Completing the treatment	6174	61	227	5886
Death	65	0	3	62
Adverse reaction	33	0	8	25
Transfer to RR-TB therapy	50	0	0	90

TPT, tuberculosis preventive therapy; IQR, interquartile range; SD, standard deviation.

**Table 3:** Characteristics of 11 tuberculosis secondary patients during follow-up time.

Characteristics	N	Bacteriologically Positive	Bacteriologically Negative or No Results	χ^2^/t	*p*-Value
Sex				0.01	0.95
Male	4	1	3		
Female	7	3	4		
Mean age, years (SD)	16.45 ± 1.57	16.25 ± 1.65	16.57 ± 1.64	0.55	0.59
City				3.80	0.28
Lianyungang	3	2	1		
Yancheng	3	0	3		
Zhenjiang	1	0	1		
Xuzhou	4	2	2		
Year				0.33	0.57
2019	3	2	1		
2020	8	2	6		
Patient type				/	
New patients	11	4	7		
Retreatment patients	0	0	0		
Complication				/	
No	11	4	7		
Yes	0	0	0		
Therapeutic regimens				0.14	0.71
2HRZE/4HR	9	4	5		
2HRZE/10HR	2	0	2		
Treatment outcome				3.93	0.05
Cure	3	3	0		
Completing the treatment	8	1	7		

SD, standard deviation; HRZE, isoniazid, rifampicin, pyrazinamide, ethambutol; HR, isoniazid, rifampicin.

## Data Availability

The data are not publicly available due to individual privacy. The data presented in this study are available on request from the corresponding author.

## Supplementary Materials

The following supporting information can be downloaded at: https://www.mdpi.com/article/10.3390/pathogens14121203/s1, Supplementary Table S1. Characteristics of TB secondary patients during follow-up time; Supplementary Table S2. Effectiveness of tuberculosis preventive treatment among 624 close contacts eligible for TPT; Supplementary Table S3. Risk factors for developing active tuberculosis among adolescent contacts with latent tuberculosis infection; Supplementary Table S4. Risk factors for developing active tuberculosis among adolescent contacts who had LTBI but did not receive TPT.

## Abbreviations

The following abbreviations are used in this manuscript:

*M.tb*	Mycobacterium tuberculosis
WHO	World Health Organization
LTBI	Latent Tuberculosis Infection
TPT	Tuberculosis Preventive Treatment
IGRA	Interferon-Gamma Release Assays
IQRs	Interquartile Ranges
IRRs	Incidence Rate Ratios
Cis	Confidence Intervals
HRs	Hazard Ratios
BCG	Bacillus Calmette–Guérin
NTM	Non-Tuberculous Mycobacteria
