# Supplementary material for: Efficacy of Isoniazid–Rifampicin Preventive Therapy in Adolescent Contacts in School Outbreaks: A Retrospective Cohort Study in Eastern China
Source: Pathogens. 2025 Nov 26;14(12):1203. doi: 10.3390/pathogens14121203 (PMC12735544; doi:10.3390/pathogens14121203)
Supplement: Supplementary file 1 [file pathogens-14-01203-s001.zip › pathogens-3991700-supplementary.docx]

Supplementary Table S1. Characteristics of TB secondary patients during follow-up time

| Characteristics | N | Bacteriological positive | Bacteriological negative or no results | χ^2^/t | *P* |
| --- | --- | --- | --- | --- | --- |
| Sex |  |  |  | 4.073 | 0.081 |
| Male | 10 | 2 | 8 |  |  |
| Female | 11 | 7 | 4 |  |  |
| Age | 21 | 9 | 12 | 0.548 | 0.59 |
| City |  |  |  | 0.058 | 0.608 |
| Lianyungang | 5 | 3 | 2 |  |  |
| Yancheng | 6 | 2 | 4 |  |  |
| Suzhou | 1 | 1 | 0 |  |  |
| Zhenjiang | 4 | 1 | 3 |  |  |
| Xuzhou | 5 | 2 | 3 |  |  |
| Year |  |  |  | 4.667 | 0.005 |
| 2019 | 11 | 6 | 5 |  |  |
| 2020 | 9 | 2 | 7 |  |  |
| 2021 | 1 | 1 | 0 |  |  |
| Patients type |  |  |  | / |  |
| New patients | 21 | 9 | 12 |  |  |
| Retreatment patients | 0 | 0 | 0 |  |  |
| Complication disease |  |  |  | / |  |
| No | 21 | 9 | 12 |  |  |
| Yes | 0 | 0 | 0 |  |  |
| Treatment regimens |  |  |  | 0.364 | 0.001 |
| 2HRZE/4HR | 16 | 8 | 8 |  |  |
| 2HRZE/10HR | 4 | 1 | 3 |  |  |
| Other regimens | 1 | 0 | 1 |  |  |
| Treatment outcome |  |  |  | 0.141 | 0.429 |
| Cure | 7 | 7 | 0 |  |  |
| Completing the treatment | 13 | 1 | 12 |  |  |
| Transfer to RR-TB therapy | 1 | 0 | 1 |  |  |

Supplementary Table S2. Effectiveness of tuberculosis preventive treatment among 624 close contacts eligible for TPT

|  | TPT | | Non-TPT | |  |
| --- | --- | --- | --- | --- | --- |
|  | N patients / N total | Follow-up time, person-years | N patients /  N total | Follow-up time, person-years | HR (95%CI) |
|  |  |  |  |  |  |
| All follow-up | 1/277 | 1088 | 10/347 | 1268 | 0.124 (0.016-0.969) |
| First three months of follow-up | 0/277 | 68 | 2/347 | 85 | 0.019 (0-1813) |
| First year of follow-up | 0/277 | 277 | 4/347 | 345 | 0.019 (0-63) |
| First two years of follow-up | 1/277 | 551 | 9/347 | 678 | 0.127 (0.017-1.079) |
| First three years of follow-up | 1/277 | 797 | 9/347 | 986 | 0.137 (0.017-1.079) |
|  |  |  |  |  |  |

TPT, Tuberculosis preventive treatment; HR, hazard ratio.

Supplementary Table S3. Risk factors for developing active tuberculosis among adolescent contacts with latent tuberculosis infection

| Characteristics | N | Patients (n) | cHR (95% CI) | aHR (95%CI) |
| --- | --- | --- | --- | --- |
| Sex of index patients |  |  |  |  |
| Male | 343 | 4 |  |  |
| Female | 281 | 7 | 2.172 (0.636-7.423) |  |
| Age of index patients | 624 | 11 | 1.272 (0.840-1.926) |  |
| City |  |  |  |  |
| Lianyungang | 165 | 3 |  |  |
| Yancheng | 128 | 2 | 0.893 (0.149-5.347) |  |
| Nanjing | 13 | 0 | 0 |  |
| Suzhou | 14 | 0 | 0 |  |
| Zhenjiang | 210 | 2 | 0.518 (0.087-3.106) |  |
| Xuzhou | 94 | 4 | 2.311 (0.517-10.341) |  |
| Year |  |  |  |  |
| 2019 | 251 | 4 |  |  |
| 2020 | 260 | 7 | 1.771 (0.518-6.061) |  |
| 2021 | 113 | 0 | 0 |  |
| Bacteriological results of index patients |  |  |  |  |
| Negative | 275 | 7 |  |  |
| Positive | 348 | 4 | 0.449 (0.131-1.532) |  |
| NA | 1 | 0 | 0 |  |
| Patients type |  |  |  |  |
| New patients | 427 | 4 |  |  |
| Retreatment patients | 197 | 7 | 3.599 (1.050-12.329) | 5.317 (1.542-18.340) |
| Sex of contact |  |  |  |  |
| Male | 330 | 5 |  |  |
| Female | 294 | 6 | 1.366 (0.417-4.475) |  |
| Age of contacts | 624 | 11 | 0.967 (0.643-1.455) |  |
| Received TPT |  |  |  |  |
| No | 347 | 10 |  |  |
| Yes | 277 | 1 | 0.124 (0.016-0.969) | 0.086 (0.011-0.678) |

cHR, crude hazard ratio; aHR, adjust hazard ratio.

Supplementary Table S4. Risk factors for developing active tuberculosis among adolescent contacts who had LTBI but did not receive TPT.

| Characteristics | N | Patient (n) | cHR (95%CI), *P* | aHR (95%CI), *P* |
| --- | --- | --- | --- | --- |
| Sex of index case |  |  |  |  |
| Male | 157 | 7 |  |  |
| Female | 190 | 3 | 1.961 (0507-7.587), 0.329 | 1.943 (0.413-9.135), 0.400 |
| Age of index case | 347 | 10 | 1.267 (0.809-1.984), 0.301 | 2.041 (1.074-3.880), 0.029 |
| City |  |  |  |  |
| Lianyungang | 110 | 3 |  |  |
| Yancheng | 76 | 2 | 0.967 (0.162-5.785), 0.970 |  |
| Nanjing | 3 | 0 | 0, 0.994 |  |
| Suzhou | 14 | 0 | 0, 0.985 |  |
| Zhenjiang | 65 | 1 | 0.582 (0.061-5.607), 0.640 |  |
| Xuzhou | 79 | 4 | 1.803 (0.402-8.080), 0.441 |  |
| Year |  |  |  |  |
| 2019 | 115 | 3 |  |  |
| 2020 | 175 | 7 | 1.592 (0.411-1.612), 0.501 |  |
| 2021 | 57 | 0 | 0, 0.983 |  |
| Bacteriological results of index patients |  |  |  |  |
| Negative | 225 | 7 |  |  |
| Positive | 121 | 3 | 0.815 (0.211-3.153), 0.767 |  |
| NA | 1 | 0 | 0, 0.987 |  |
| Patients type |  |  |  |  |
| New patients | 268 | 8 |  |  |
| Retreatment patients | 79 | 2 | 4.888 (1377-17.353), 0.014 | 1.929 (1.546-22.744), 0.009 |
| Sex of contact |  |  |  |  |
| Male | 177 | 4 |  |  |
| Female | 171 | 6 | 1.569 (0.443-1.560), 0.485 | 1.454 (0.398-5.310), 0.571 |
| Age of contact | 347 | 10 | 0.983 (0.654-1.479), 0.935 | 0.649 (0.361-1.167), 0.149 |
